# Thyroid Ectopia in the Euthyroid Patient: A Rare Case Discussion

**DOI:** 10.7759/cureus.116636

**Published:** 2026-09-21

**Authors:** Melissa Cummins, Levi Stevens

**Affiliations:** 1 Otolaryngology, West Virginia University Hospitals, Martinsburg, USA; 2 Medicine, West Virginia School of Osteopathic Medicine, Lewisburg, USA

**Keywords:** base of tongue, congenital spinal abnormalities, ectopic thyroid, euthyroid, thyroglossal duct cyst

## Abstract

Base-of-tongue masses are difficult to differentiate based on imaging alone. The vast majority of masses in this region are squamous cell carcinomas, necessitating prompt and thorough evaluation. However, particularly in deep, midline, submucosal distributions, congenital masses are an important differential to consider due to the embryological correlates of aberrant structures in this location. For patients in whom ectopic thyroid is suspected, it is imperative to discern whether functional orthotopic thyroid tissue is present. Most patients with thyroid ectopia have some extent of thyroid dysfunction, the degree of which will impact definitive management. Many patients are asymptomatic, while those who do present with symptoms report largely nonspecific findings such as dysphagia and dry cough. We present the case of a 60-year-old female patient who presented to an otolaryngology clinic for evaluation after a base-of-tongue mass was noted incidentally on imaging after a fall. Initial workup included flexible laryngopharyngoscopy for direct visualization of the mass, followed by biopsy revealing ectopic thyroid tissue.

## Introduction

In patients presenting with base-of-tongue masses, the differential is broad and encompasses both malignant and benign lesions. Base-of-tongue masses are difficult to differentiate based on imaging alone due to their nonspecificity [1]. Importantly, the vast majority of malignant masses in this region are squamous cell carcinomas, necessitating prompt and thorough evaluation [1]. However, particularly in deep, midline, submucosal distributions, congenital masses are an important differential to consider due to the embryological correlates of aberrant structures in this location. In rare cases, base-of-tongue masses may represent ectopic lingual thyroid tissue, rather than malignancy.

The typical migration of the primordial thyroid begins during the fourth week of gestation at the foramen cecum near the base of the tongue and proceeds caudally before reaching the pretracheal space just ventral to the thyroid cartilage by the seventh week of gestation [2]. This path of descent courses along the thyroglossal duct, an embryologic structure that obliterates during typical development [2]. Additional thyroid tissue, parafollicular cells responsible for maintenance of calcium homeostasis through the release of calcitonin, is derived from the ultimobranchial bodies, which originate from lateral outpouches of the fourth pharyngeal arches and migrate to fuse with the primordial thyroid [3]. 

Although uncommon, ectopic thyroid tissue is identified in one per 100,000 to 300,000 patients, with a strong female predominance (80%) [4]. Although the mechanism driving female predominance is unclear, it has been theorized that hypermetabolic and hypometabolic states such as autoimmune-associated thyroiditis, pregnancy, or menopause may contribute to increased thyroid hypertrophy or symptomaticity and therefore detection among female patients [5]. Among patients with hypothyroidism, ectopic thyroid tissue is more common and identified in one per 4,000 to 8,000 patients [6]. Due to its dual embryologic origin, there are a variety of phenotypes typical of patients who experience aberrancy in thyroid anatomy [3]. Ectopic thyroid tissue can present in many locations, most commonly between the foramen cecum and the diaphragm as a result of failed midline descent from the foramen cecum [3]. Less commonly, ectopic thyroid tissue has been reported in remote or lateral structures including the iris, female genital tract, gastrointestinal system, and pituitary gland, with aberrant migration of ultimobranchial bodies proposed as a possible mechanism [3-4]. Lingual thyroid tissue is the most common variant of ectopic thyroid, and most patients are asymptomatic and identified incidentally [4]. Conversely, for patients with significant comorbid hypothyroidism, detection is often possible through routine newborn screenings [7]. However, among patients with ectopic thyroid tissue, 10-30% will have coexisting orthotopic thyroid tissue and may present with euthyroid function [6]. Regardless of the presence of orthotopic tissue, it is important to consider the possibility of malignant transformation for any patient with ectopic thyroid tissue, although this occurs in less than 1% of cases [4]. 

For patients in whom ectopic thyroid is suspected, it is imperative to discern whether functional orthotopic thyroid tissue is present. Most patients with thyroid ectopia have some extent of thyroid dysfunction, the degree of which will impact definitive management [6]. 

## Case presentation

We present a 60-year-old female patient who presented to our otolaryngology clinic for evaluation of a base-of-tongue mass incidentally noted on imaging after a ground-level fall. Past medical history was notable for narcolepsy, type 2 diabetes mellitus, history of two mitral valve replacements, a remote history of alcohol and tobacco use disorders, and a remote history of treatment for hypothyroidism. At the time of presentation to the emergency department, the patient was asymptomatic, with no evidence of dysphagia, odynophagia, hemoptysis, or globus sensation. 

Imaging at the time of the initial encounter incidentally noted developmental nonunion of the posterior arch of C1 (Figure 1) and a well-circumscribed, hyperdense, 2.6 x 2.3 x 1.7 cm mass in the base of the tongue, just superior to the epiglottis (Figure 2). The patient was referred for outpatient evaluation by otolaryngology to further delineate the origin of the mass. Differential diagnosis included thyroglossal duct cyst, squamous cell carcinoma, ectopic thyroid tissue, and venous malformation.

**Figure 1:**
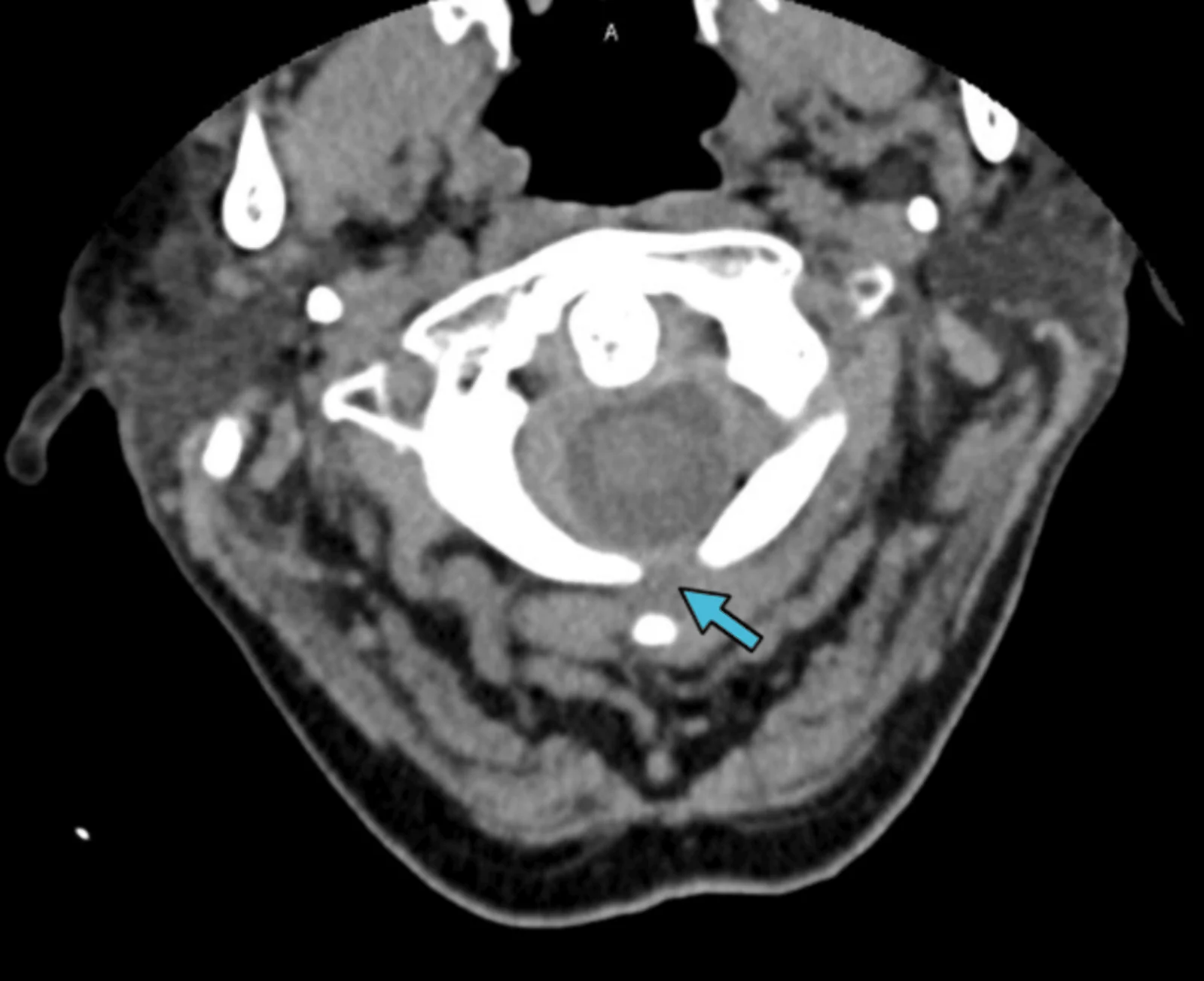
Noncontrast CT scan demonstrating posterior atlas arch nonunion (blue arrow) in the axial plane

**Figure 2:**
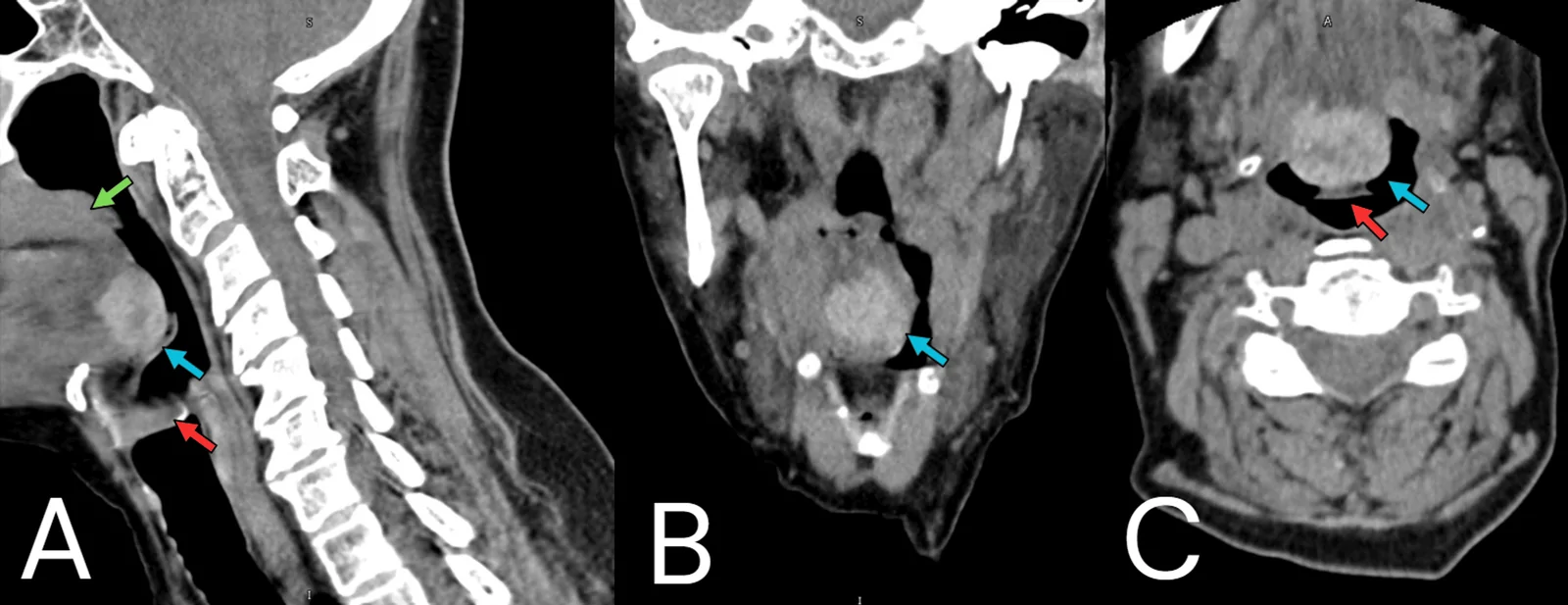
Noncontrast CT scan demonstrating 2.6 x 2.3 x 1.7 cm mass (blue arrows) at the base of the tongue, located superior to the epiglottis (red arrows) and inferior to the soft palate (green arrow) in sagittal (A), coronal (B), and axial (C) planes

At the time of assessment in clinic nine days after initial presentation, the patient endorsed frequent throat clearing when talking for prolonged periods of time, but noted no other symptoms. On exam, the patient was afebrile and normotensive. There was no palpable cervical lymphadenopathy, thyromegaly, or masses of the salivary glands or neck. In-office flexible laryngopharyngoscopy for direct visualization of the mass demonstrated a well-circumscribed, rounded, exophytic mass at the base of the tongue without evidence of mucosal erosion. The nasopharynx, oropharynx, and hypopharynx were otherwise unremarkable. 

Due to the patient’s history of tobacco and alcohol exposure and the inability to fully characterize the mass based upon appearance, biopsy was recommended to rule out malignancy. Routine screening of the thyroid-stimulating hormone (TSH) was 3.57 (reference: 0.4-4.2) two weeks prior to completion of the biopsy. Tissue samples collected from the mass on direct laryngoscopy, completed 50 days after initial presentation, demonstrated diffuse immunohistochemical positivity for thyroid transcription factor-1 (TTF-1) and follicular arrangement on frozen section analysis, consistent with benign thyroid tissue. At postoperative follow-up 69 days after initial presentation, review of the CT imaging obtained at the time of initial presentation demonstrated absence of thyroid tissue in the pretracheal region (Figure 3). On exam, the surgical site was appropriately healed with evidence of fibrination. Due to the absence of functioning eutopic thyroid tissue in the thyroid bed and the asymptomatic status of the patient, excision of the ectopic thyroid was deferred, and routine follow-up was established annually.

**Figure 3:**
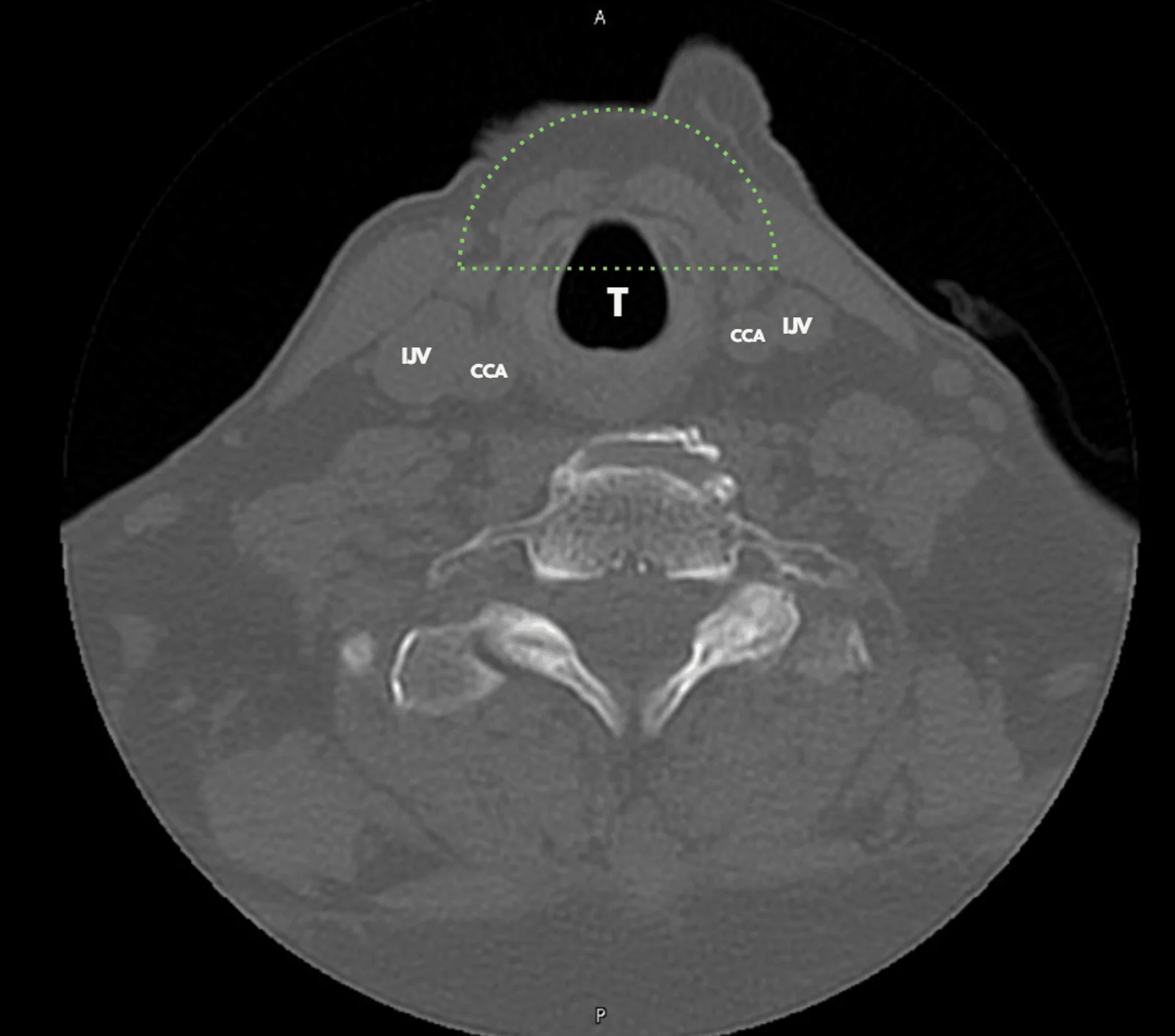
Axial CT cervical spine without contrast demonstrating the absence of eutopic thyroid in the infrahyoid pretracheal region (dotted green semicircle) Landmarks noted include the trachea (T), common carotid arteries (CCA), and internal jugular veins (IJV)

## Discussion

Differentiation of masses in the base of the tongue region presents a unique challenge in that they are difficult to characterize using imaging alone [1]. When suspicion for congenital thyroid aberrancies is high, modalities such as technetium scanning can provide valuable diagnostic information without subjecting patients to the risks inherent in biopsy and can help exclude a vascular lesion before biopsy when appropriate. Particularly salient clues include a history of abnormal thyroid function or the absence of identifiable eutopic thyroid tissue on imaging. However, particularly for patients with borderline or fluctuating thyroid function, this diagnostic algorithm becomes less clear, as ectopic thyroid is far less common in euthyroid patients. For example, in our case, despite not having received treatment for hypothyroidism for four months, routine screening of TSH was 3.57 (reference: 0.4-4.2) two weeks prior to completion of the biopsy. Due to euthyroid status, further thyroid function testing was ordered at the time of surgery or postoperatively. Upon further review, additional historical medical documentation also revealed a remote history of neck ultrasound in which the thyroid was unable to be visualized. However, no further workup was initiated, and the finding was likely attributed to technique rather than anatomy. Further exploration of the nonvisualized thyroid tissue at that time, over a decade ago, may have prevented the need for biopsy in this case. Together, these findings highlight the importance of monitoring thyroid function over time as well as the utility in characterizing thyroid ectopia when it is reasonably plausible, particularly in cases where the thyroid is not visualized on routine scans, or where associated findings such as hypothyroid state are present.

Additionally, the presence of two incidentally identified midline anomalies, congenital atlas arch hypoplasia and lingual thyroid, is noteworthy in this patient. Transcription factors such as forkhead box protein E1 (FOXE-1) are essential drivers of typical caudal migration of the primordial thyroid, as well as simultaneously driving development of midline axial structures including the cervical spine [8-10]. The role of FOX-E1 in both midline axial development and caudal thyroid migration has been posited as a potential mechanism for the co-occurrence of ectopic thyroid with midline axial anomalies in some patients [8-10]. Of note, there is also a significantly greater likelihood of heart defects in patients with congenital hypothyroidism, although the mitral valve insufficiency in this patient is presumed to be of rheumatic origin [8-10]. When evaluating the origin of base-of-tongue lesions, the presence of additional midline defects or an associated hypothyroid state are additional indications that lend greater merit to the consideration of thyroid aberrancy as a leading differential diagnosis.

## Conclusions

Although base-of-tongue lesions are not commonly attributable to ectopic thyroid tissue, there are several conditions that warrant heightened clinical suspicion. These include histories of hypothyroidism, associated midline defects including those of the heart and axial skeleton, and prior difficulties with routine imaging. The appropriate clinical constellation should prompt providers to consider further diagnostic imaging, such as scintigraphy, to identify ectopic thyroid tissue and forego invasive diagnostic procedures where possible. Importantly, in symptomatic patients for whom ectopic thyroid tissue is identified, it is imperative to discern whether eutopic thyroid tissue is present. For the vast majority of patients, ectopic thyroid tissue, though frequently insufficient to reach a euthyroid state, will be the only functional thyroid tissue present. In these cases, it is important to discuss the risks and benefits of excision, including the absence of autologous thyroid function, the risk of malignant transformation, and the risks inherent to the excision itself. For many patients, conservative management and surveillance of thyroid function is appropriate and imposes minimal risk.
